# Meeting the Needs of Families Involved in the Child Welfare System for Parental Substance Abuse: Outcomes From an Effectiveness Trial of the Families Actively Improving Relationships Program

**DOI:** 10.3389/fpsyg.2021.689483

**Published:** 2021-07-02

**Authors:** Lisa Saldana, Jason E. Chapman, Mark Campbell, Zoe Alley, Holle Schaper, Courtenay Padgett

**Affiliations:** Oregon Social Learning Center, Eugene, OR, United States

**Keywords:** FAIR, opioid, methamphetamine, child welfare, mental health, evidence-based practice, parent

## Abstract

Limited evidence-based practices exist to address the unique treatment needs of families involved in the child welfare system with parental substance abuse. Specifically, parental opioid and methamphetamine abuse have increased over the last decade, with associated increases of families reported to the child welfare system. The Families Actively Improving Relationships (FAIR) program was developed to address the complexities of these families. Evidence-based strategies to address the interrelated needs of parents—including substance abuse and mental health treatment, parent skills training, and supportive case management to improve access to ancillary needs—are integrated in an intensive community outpatient program. This study examined the clinical effectiveness of FAIR when delivered in a Medicaid billable outpatient clinic. Parents (*n* = 99) were randomized either to the immediate FAIR condition or to the Waitlist (WL) condition, using a dynamic wait-listed design, with all parents provided the opportunity to eventually receive FAIR. Outcomes show statistically and clinically significant reductions in parental opioid and methamphetamine use, mental health symptoms, and parenting risk, and improvements in stability in parents receiving FAIR. Providing services to families who require travel in excess of 20 miles for sessions has challenging implications for program costs under a Medicaid structure. Study outcomes highlight the need for policies to support funding of intensive family-based programs.

## Introduction

During 2019 across the United States, the child welfare system (CWS) received 4.4 million referrals for child maltreatment involving ~7.9 million children (DHHS, 2021). The rate of referral rose from 52.3% in 2015 to 59.5% in 2019, with a 5.8% increase in referrals that were screened-in for services during this same period. The majority of children were exposed to child neglect (75%), and the majority of perpetrators were parents (91.4%). Similarly, following a decade of steady decline in the number of children in foster care, rates began to rise nationally in 2012, with an increase of over 10% by 2016 (DHHS, 2019). During this period, there was a parallel increase in the number of CWS reports related to parental drug abuse, prompting formal federal tracking of these referrals beginning in 2015. Since then, rates of parental drug abuse continue to rise, with drug abuse risk factors greatest for children under 1 year old (DHHS, 2021).

States' rates of child maltreatment and rates of parental substance use vary, but across the nation, 36 states have experienced a significant increase in CWS caseloads (DHHS, 2019). These increases are simultaneous with the rise in the nationwide opioid epidemic, with the CWS being particularly impacted by its effects (Crowley et al., 2019). In parallel, particularly in the western states, methamphetamine use has shown marked increase in populations that use opioids (Ellis et al., 2018), and the co-occurrence of opioids and methamphetamine is rising (Volkow, 2020). Children whose parents are referred for methamphetamine abuse are more likely to enter into foster care, and less likely to reunify home than children of parents referred for other reasons (Akin et al., 2015). Despite these notable challenges, few evidence-based behavioral interventions have been developed specifically to address the complex needs of families involved in the CWS where opioid and/or methamphetamine abuse is the primary referring problem.

**Figure 1:**
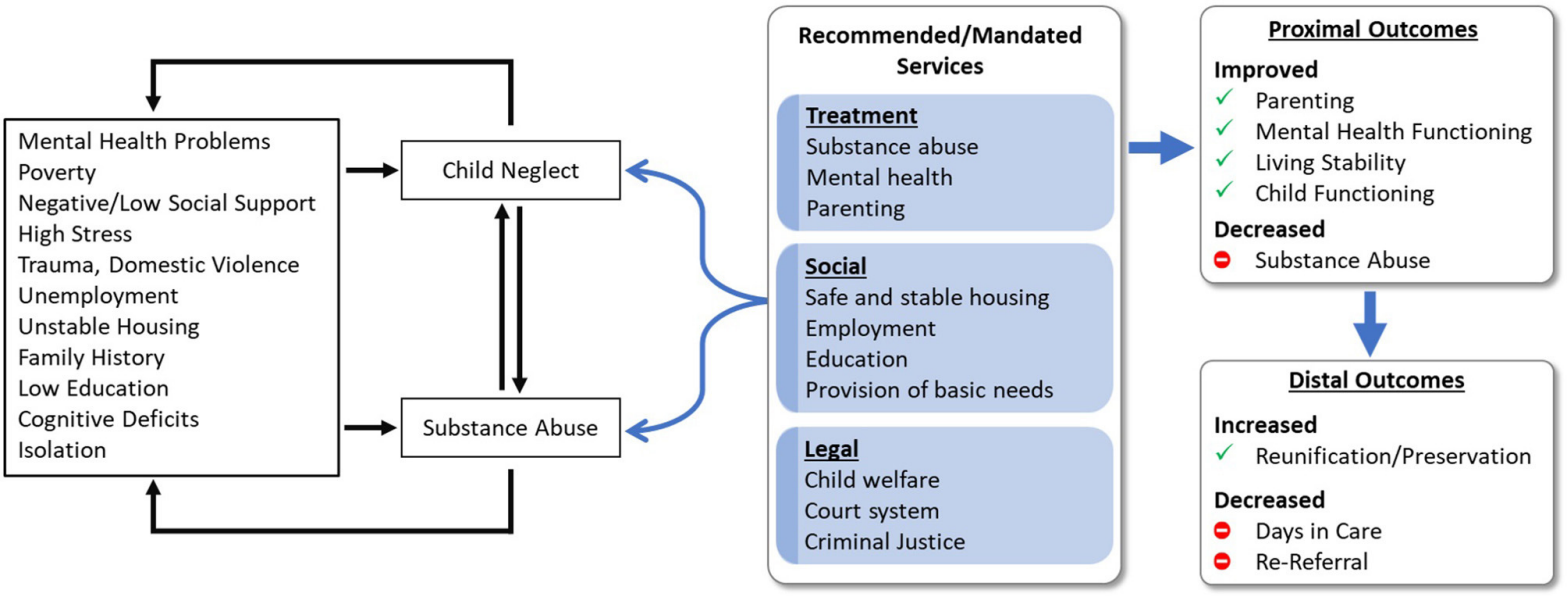
Logic model for the Families Actively Improving Relationships (FAIR) program for parents involved in the child welfare system for parental substance abuse and child neglect.

To fill this critical gap, the Families Actively Improving Relationships (FAIR) program was developed (Saldana, 2015). The goal of FAIR is to provide evidence-based practices (EBPs) within the environment in which parents live and function, to a population that is extremely difficult to engage. FAIR addresses the shared correlates of, and interplay between, substance abuse, mental health, and parenting needs, and it operates from a treatment plan that addresses a comprehensive set of CWS goals (Figure 1). Specifically, FAIR aims to address the gap between the known correlates that drive both parental substance abuse and child neglect and receipt of services that are needed to achieve both proximal and distal positive outcomes.

Using a well-specified behavioral approach, FAIR treatment is individualized to fit the unique circumstances and needs of families presenting with opioid and methamphetamine use disorders. FAIR clinicians coordinate with CWS staff to ensure that parents are meeting their CWS treatment plan goals. Parents are incentivized for working toward their treatment goals that increase child safety and permanency. FAIR allows for delivery of EBP within a flexible environment including meeting times and places (e.g., home, shelter, tent, park) and in the community where parents have the opportunity to practice success (e.g., store, school, playground). Similar to other family-based EBPs, such as Multisystemic Therapy (MST; Henggeler et al., 2009), the FAIR team is available 24/7 for on-call support and ongoing engagement strategies.

**Figure 2:**
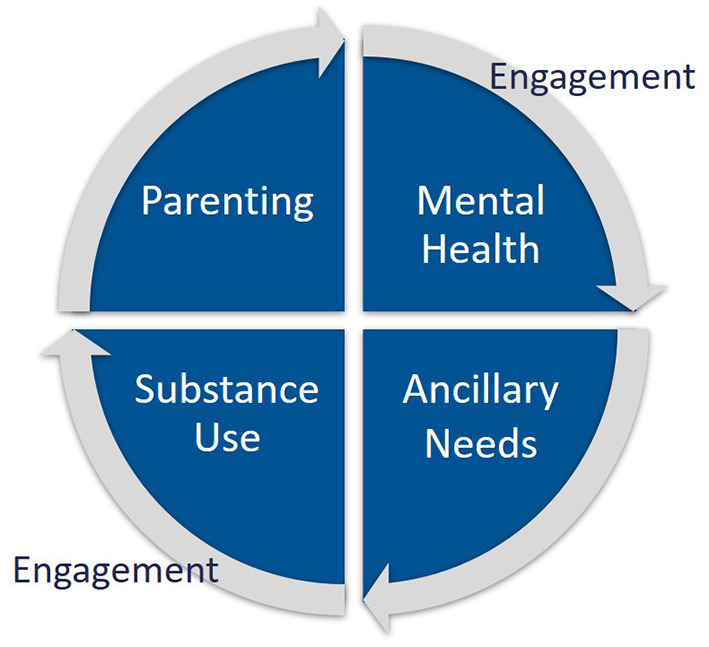
Core treatment components of the FAIR program, supported by ongoing active engagement.

FAIR involves four major treatment components, supported by ongoing purposeful engagement (Figure 2): (1) Substance use treatment including contingency management and positive reinforcement, frequent urinalysis, relationship building, day planning, skill building in creating healthy environments and peer choices, and refusal skills; (2) Mental health treatment including cognitive behavioral strategies, developing healthy coping skills, emotion regulation skills, exposure therapy, and referral for medication management; (3) Parent management training including parenting skills, nurturing and attachment, reinforcement, emotion regulation, supervision, structure, non-harsh discipline, and nutrition; and (4) Resource building and provision of ancillary supports including assistance with securing housing, education, employment, and support with court and CWS attendance, and other probationary requirements. Traditionally, each of these treatment components are delivered in a siloed manner, with multiple providers. This traditional arrangement often requires parents to balance a complex treatment schedule and find transportation to multiple service settings. Moreover, many of the services that are accessible to families are not evidence-based or designed with their unique treatment needs in mind (e.g., childcare, competing court requirements).

FAIR provides an action-oriented approach to treatment. Unlike a typical treatment session, parents and counselors engage in hands-on problem-solving and solution-focused goal setting. In this way, the FAIR model is perceived as supportive and useful to parents, while counselors are able to role-play, model, and skill build with parents during real-world scenarios (Cruden, Crawford, and Saldana, submitted manuscript). FAIR counselors also leverage a team resource builder to identify incentives and prosocial community activities that can be used throughout treatment. Tailoring incentives to the individual needs of families, while adhering to the key FAIR treatment components, allows parents to experience evidence-based services that are meaningful to their daily lives, in the environments in which they live. Incremental feedback on behaviors is provided through behavioral reinforcement strategies in ways that feel natural to parents, including text messages from counselors about a job well-done, a small goal set, or an observation of progress. Counselors also learn the preferences of individual parents and might provide engagement strategies such as a favorite warm beverage for an early morning session, or a sandwich when meeting on a lunch break.

The use of home and community-based treatment and contingency management strategies are consistent with other programs that have evidenced promise in addressing the needs of parents involved in the CWS with substance abuse. Family Behavior Therapy (FBT; Donohue et al., 2014) demonstrated success in the treatment of mothers referred to the CWS, with treatment showing the greatest effectiveness for mothers whose children were not drug exposed. Mothers randomized to FBT also increased days employed. An adaptation of Multisystemic Therapy (Henggeler et al., 2009) called Building Stronger Families (MST-BSF; Swenson and Schaeffer, 2018) has been piloted in a quasi-experimental matched design showing promise for the reduction of maternal substance use and aggression toward children (Schaeffer et al., 2013). Similar to FAIR, these programs help overcome barriers that have long been identified for parents who are involved in the CWS (Young et al., 1998), including lack of childcare, inadequate support from family and friends, copayments, and time allowed away from work (Rockhill et al., 2008). Parents often require ancillary services, including employment assistance, food security, housing, and transportation (Choi and Ryan, 2007). Not surprisingly, receipt of these basic services enhances caregiver ability to start and complete substance abuse treatment (Smith and Marsh, 2002; Greenfield et al., 2007), which ultimately facilitates reunification (Grella et al., 2009).

Using intervention strategies intentionally focused on overcoming the barriers that parents involved with the CWS for substance abuse and neglect experience, the FAIR program has demonstrated positive outcomes for mothers randomized to receive FAIR, including reductions in substance use, cravings, mental health symptoms, and parenting stress and improvements in child behavior (Saldana, 2015). Due to the positive outcomes obtained from these original feasibility and randomized pilot trials, the CWS requested ongoing availability of the FAIR program and, thus, FAIR moved from a research funded environment to a Medicaid billable free-standing clinic. Referrals were made directly by child welfare case workers and/or parent self-referral. Moreover, services were extended to fathers as well as mothers. As community clinicians were hired to provide services and supervision and the program grew to be independent from the original research trials, there was an opportunity for the current effectiveness trial, under real-world conditions.

Due to the perceived benefits of the FAIR program, the local CWS agreed to provide referrals to the current study under the condition that all parents would have the opportunity to receive FAIR. Because of the nature of CWS involvement and federal timelines imposed for potential CWS treatment plan completion (i.e., 18 months), along with the length of treatment for FAIR (i.e., ~9 months), a traditional randomized clinical trial was not plausible. A dynamic wait-listed design (Brown et al., 2006), described in more detail below, was employed to maintain rigor, but provide the opportunity for all to receive the experimental intervention. Effectiveness trial primary hypotheses included: Parents receiving FAIR would experience reductions in (1) parental substance abuse, in particular opioid and methamphetamine use, (2) parental mental health problems, and (3) parenting risk. Further, it was hypothesized that these parents would experience improvement in ancillary needs and stability (i.e., days employed, more stable housing). In addition to these clinical effectiveness questions, this trial allowed the opportunity to assess the conditions under which such a program can be sustained in the real world—an economic analysis examined the feasibility of providing this comprehensive, integrated program within a Medicaid billable environment.

## Materials and Methods

### Study Design

The study was designed to accommodate several ethical and practical considerations. Most critically, participants could not be randomized to a traditional services condition due to the desire of the local CWS to have the opportunity for every referred parent to eventually receive FAIR. That is, all participants needed to be able to have access to the experimental intervention. However, in order to evaluate FAIR, it was important to obtain comparison data from traditional services. To address this, the study was designed as a restricted case of a dynamic wait-listed design, with each participant potentially having repeated outcome measurements during a longitudinal waitlist phase and, upon initiating FAIR, longitudinal measurements during the FAIR phase. From this, a key feature of the design is that intervention condition was not a single status for each participant; rather, it changed over time if participants transitioned from the waitlist to FAIR. Regardless of condition, a baseline assessment was scheduled within 72 h of a parent agreeing to be in the study. All participating parents were assessed for 20 to 24 months post-final baseline. This variation in the final assessment time-point was due to the grant period ending—all participants were assessed as close to their 24-month period as feasible (hereafter referred to as 24-month).

Another design consideration was the need to be able to move parents off of the waitlist, if they desired, as soon as openings became available. This was necessary to address the rapid timeline imposed by federal mandate (Adoption and Safe Families Act, 1997) that parents have to complete CWS treatment plans and establish permanency goals. While it was anticipated that many parents randomized to the waitlist (WL) would engage in traditional treatment (as they were encouraged to do at the time of randomization), and therefore not be interested in FAIR when offered an opening, in fact the majority chose to participate and to do so quickly. This resulted in only 17 of 99 participants having multiple measurements in the waitlist phase, of which only ten were assessed more than twice. As such, it is not possible to compare changes during the waitlist phase to changes during the FAIR phase. Despite this limitation, with a non-trivial percentage of the total observations in the waitlist phase (10%; 43 of 448 observations), these data were retained in the final analyses (as detailed in the Data Analysis Strategy). Further, as is encouraged for parents randomized to the WL condition, eight parents chose to engage in alternative services and not ever engage in FAIR. Outcomes related to these parents provide a very small, but valuable comparison for discussion.

### Referrals, Consent, and Randomization

Referrals were made directly to the clinical coordinator who screened for eligibility criteria. When the parent met eligibility criteria, the coordinator set up a meeting with the parent to describe the study and review the Oregon Social Learning Center IRB-approved informed consent and protocol.

Eligibility criteria mirrored the FAIR program real-world criteria and included: (a) identification of child neglect as determined by child welfare, (b) a finding or other indication of parental substance abuse, (c) child(ren) remaining in the home or having a plan for reunification (i.e., termination of parental rights had not occurred), and (d) the parent was English speaking. Parents must have reported problems with substances other than THC and/or alcohol alone. The reason for these exclusionary criteria was the contingency management approach to treatment: alcohol alone was too difficult to reliably detect, and THC is detected by urinalysis for several days to weeks after last use; in both cases, it was not feasible to provide immediate reinforcement for certain level of evidence. There were no exclusionary criteria related to parental age, race/ethnicity, or child age. Throughout the study, 124 parents were referred and screened, 108 of whom were eligible. Of these, 99 consented to participate.

Following baseline assessment, participants were randomized to either FAIR or traditional treatment as part of the WL condition, with the exception of the first cases (*n* = 5) that were assigned to FAIR to fill counselor caseloads and provide an opportunity for a waitlist period. Parents randomized to FAIR were referred to the FAIR intake assessor to schedule an appointment. Parents initially allocated to WL were offered a list of referrals for traditional services and assistance in contacting them. Regardless of the initial study condition, the referring caseworker was notified so that additional referrals could be made as necessary.

For parents who were initially allocated to WL, their later invitation to the FAIR condition was based on the availability of an opening on a caseload. Once those assigned to FAIR terminated the intervention (i.e., either treatment completion or drop-out), thereby creating an opening on the FAIR caseload, if the next referred parent was not randomized to FAIR, the next parent on the waitlist was contacted to determine if (s)he was engaged in traditional services and if (s)he was still interested in receiving FAIR. Importantly for the adapted dynamic wait-listed design, parents were not notified when they were “next in line” in order to avoid the potential of them not engaging in traditional services based on the hope that a FAIR slot would soon be available. The final sample included *n* = 59 parents randomized to FAIR, *n* = 32 initially randomized to WL who then later transitioned to FAIR, and *n* = 8 who were randomized to WL and decided never to receive FAIR.

#### Participants

Of recruited parents, 74 were mothers and 25 were fathers. Of these, 47 mothers and 20 fathers reported being non-Hispanic and White/Caucasian, 5 mothers reported being Hispanic and White/Caucasian, 13 mothers and 2 fathers reported being non-Hispanic and multi-racial, and 5 mothers and 2 fathers reported being Hispanic and multi-racial. The average age of participants at baseline was 31.34 years old (range = 15–51 years), and their average number of children was 2.41 children (range = 0 to 6). One mother reported 0 children at the time of her baseline assessment because she was pregnant with her first child; a second mother also was referred when pregnant, but she already had given birth to other children. Parents primarily never were married (57%) or married (19%). The majority of parents were referred for methamphetamine use (71%), with others referred for co-occurring opioid use (24%) or opioids alone (5%).

### Treatment Conditions

#### FAIR

The FAIR program is an intensive community-based treatment model that integrates components of two evidence-based behavioral interventions: (1) Parent Management Training-Oregon (PMTO, recently renamed Generation PMTO; Forgatch and Patterson, 2010), developed at the FAIR home institution, to increase parenting skills, and teach and support positive family interactions, and (2) Reinforcement Based Therapy, a community reinforcement approach of contingency management (RBT; Jones et al., 2005) to address adult substance use. The FAIR home and community-based delivery of care, alongside the inclusion of indigenous supports, is consistent with other EBPs for family-based problems (e.g., Functional Family Therapy; Alexander and Parsons, 1982; Multidimensional Family Therapy; Liddle et al., 2018), and the FIT assessment was adapted for use, with permission, from Multisystemic Therapy (Henggeler et al., 2009). FIT assessments help counselors to understand the interconnection of challenging behaviors and areas of strength, to identify the best point of intervention (Saldana and Henggeler, 2006).

A key component of the FAIR program is the use of the FAIR store. Parents receive FAIR bucks as reinforcers for positive treatment gains. This contingency management system is used to reinforce negative urinalysis, the use of positive parenting strategies, completion of applications toward achievement of ancillary goals, or other positive steps toward goal achievement. FAIR bucks are delivered liberally to recognize the incremental gains that parents make. The FAIR store is purposefully stocked with donated goods provided through deliberate outreach by a resource builder. Store items include adult and child seasonal clothing, interactive games and toys, hygiene supplies and toiletries, household goods, and child safety equipment. Donations are sought in order to ensure a sustainable supply of parent-targeted incentives that can be accessed without fiscal support. In addition, community resources are sought to help support parents in engaging in prosocial community activities (e.g., passes for swimming lessons, scholarships for child summer camps). Parents are able to spend their FAIR bucks on incentives that help them support their individual and parenting goals. Through the use of FAIR bucks, parents learn that it is their “job” to make prosocial choices for themselves and their families, and that doing so enables them to progress toward their goals. Moreover, the FAIR store provides the opportunity for counselors to work with parents on issues of budgeting, prioritizing needs, and selection of developmentally appropriate supplies.

The FAIR team includes counselors, a clinical supervisor, and a resource builder. A single supervisor can support up to 7 counselors, with a part-time resource builder serving families across counselors. Because the majority of services are delivered in the community, the FAIR clinic space is minimal and includes a shared team office, supervisor office, FAIR store, and a session room for parents who want to meet at the clinic (e.g., unhoused parents during inclement weather).

The principles for counselor-family interactions are based on elements that have demonstrated success in engaging caregivers in EBPs such as PMTO (Forgatch and Patterson, 2010) and KEEP (Chamberlain et al., 2008). Counselors engage parents in their natural home and community environments and reinforce the use of prosocial strategies to accomplish the parents' goals. Sessions are action oriented and often involve role plays and hands-on teaching of new skills in the environments in which they will be used, followed by practice assignments. Counselors are trained to find opportunities in every interaction to reinforce parents for positive gains (Saldana, 2015). Counselors maintain frequent (at least monthly) contact with child welfare caseworkers to provide updates on progress and to ensure that treatment includes the goals targeted on the CWS treatment plan.

#### Traditional Treatment Services

Parents who were randomized to the WL condition were encouraged to seek traditional therapy services offered in the community. Of the 40 parents that were initially allocated to WL, seven received some level of mental health treatment. Specifically, participants received: Individual therapy (*n* = 5 parents; range 2–42 visits), family therapy (*n* = 4 parents; range 1–14 visits), and group therapy (*n* = 3 parents; range 2–15 visits). Of the three parents receiving group therapy, one also received individual therapy, one also engaged in family therapy, and one engaged in all three forms of therapy. One parent received both individual and family therapy, but no group therapy.

In addition, substance abuse-specific services were received. Four individuals who received traditional therapy also received substance abuse treatment, for a total of seven parents who received substance abuse treatment (range 1–77 days; average of 24 days). Two parents participated in a day treatment program (for 3 and 100 days) and one parent received 1 day of inpatient treatment. One parent reported 15 residential treatment attempts for a total of 102 days. Three parents reported attending a substance use disorder support group and two reported attending a recovery/rehabilitation group. Finally, 10 participants reported attending NA/AA groups (range 1–64 times; average 15.7 times). Of these, five were participants who reported some type of substance abuse treatment service also.

### Data Collection Procedures

In-person assessments were collected at Baseline, 4-, 8-, 16-, and 24-months. All assessments were collected by trained research assessors at times that were convenient and in the parents' homes or places of their choosing, including the research office. Brief monthly assessments with parents were collected via telephone for the first 15 months post-baseline. As expected, repeated attempts often were needed to arrange in-person appointments and complete monthly data collection. In instances where contact was not made successfully via telephone, the research team made efforts to locate the parents at home, work, or other community settings. The participating parents were compensated for their time with gift cards to commonly utilized stores or gas stations. Payments were $100 for each full assessment battery time-point, and $20 for each monthly phone assessment. Across data collection waves, completion rates were high (Baseline = 100%; 4-month = 88%; 8-month = 89%; 16-month = 81%; 24-month = 93%; monthly phone calls = 67%). As part of the study design, participants could have repeated measurements in the WL phase and/or the FAIR phase, and as such, there could be more than five measurements per participant.

#### Data Management

Assessment measures were programmed into the SNAP Survey software package, and all responses were entered directly into a computer during the assessments. The SNAP program allowed for field parameters to be set to ensure that items were not missed and that invalid codes were not entered. Using this system, data quality and integrity was ensured from the point of collection. Changes to data due to entry errors could only be made by the data manager. The measures for each interview were linked by a participant identification number. Data were transferred to the secure server either through an encrypted upload system, or directly from an encrypted external drive depending on internet availability. Data immediately were exported to SPSS files for cleaning, verification, and processing. Data from this trial are not publicly available, but requests for trial data can be made to the first author.

### Full Assessment Battery Measures

Assessments were collected using web-based data collection software, with an offline option, to aid in reliable assessments under varying technology conditions. Paper and pencil options were available as back-up if necessary.

#### Parental Substance Abuse

##### The Addiction Severity Index (ASI)

The ASI (McLellan et al., 1980) is a standardized tool for evaluating days, amount, and kind of substance used, as well as psychosocial correlates of use including family, housing, and employment outcomes. This self-report assessment includes use, behaviors, and correlates across the lifespan as well as in the last 30 days. The ASI has strong psychometrics and is commonly used in research and clinical practice. The parent self-reported methamphetamine, opioid, and IV drug use outcomes were dichotomous, reflecting any reported use in the past 30 days.

#### Parenting Risk

##### The Parenting Stress Index (PSI)

The PSI (Abidin, 1995) is a 101-item questionnaire developed to assess the level of stress in a parent–child system. The PSI was developed on the theory that the total stress a parent experiences is a function of certain salient child characteristics, parent characteristics, and situations that are directly related to the role of being a parent. Psychometric properties are adequate, and higher PSI scores reflect higher levels of parent-reported stress. Scores at or above the 85% are considered clinically significant.

##### The Brief Child Abuse Potential Inventory (BCAP)

The BCAP (Ondersma et al., 2005) is a validated 33-item self-report questionnaire that includes six subscales: distress, family conflict, rigidity, happiness, feelings of persecution, loneliness, and financial insecurity. The BCAP is a strong predictor of neglectful parenting. Higher scores reflect a greater risk for child neglect.

#### Parental Mental Health

##### The Trauma Symptom Inventory (TSI)

The TSI (Briere, 1995) is a 100-item questionnaire that assesses posttraumatic symptomatology and psychological functioning. Subscales include assessment of anxiety, arousal, anger, intrusive thoughts, defensive avoidance, dissociation, sexual concerns, impaired self-reference, and tension reduction behavior. Validity scales evaluate inconsistent responding. The TSI has demonstrated strong psychometric properties with a range of populations. The present study considers anxiety T-scores, with higher scores reflecting higher levels of anxiety, as well as a dichotomous clinical-level score for the anxiety subscale, and a clinical-level trauma score across any subscales above the clinical threshold.

##### The Beck Depression Inventory (BDI)

The BDI (Beck and Steer, 1993) is a well-established, 21-item self-report measure, widely used with acceptable reliability and validity. Participants are asked to choose one of four statements that range from positive to depressed feelings about life in the past week. Higher scores reflect higher levels of depression symptoms.

#### Individual Characteristics

##### Demographics Questionnaire

The demographics questionnaire queried parents about their personal demographics and the characteristics of their children. It only was asked at baseline.

### Monthly Assessments

#### The Parent Daily Report (PDR)

The PDR (Chamberlain and Reid, 1987) is a 31-item questionnaire completed by caregivers about child behaviors in the previous 24 h. Parents reported whether or not any of the problem behaviors occurred and if the occurrence was stressful for the parent. The PDR has demonstrated adequate psychometric properties (Keil, 2007). The PDR has been adapted for the FAIR treatment trials with additional items to query about parental cravings and mental health concerns. Parents reported “in the last 24 h” how often they had thought about using drugs, how strong their cravings were at their most severe point, how difficult it would have been to resist using drugs if available, overall rating of cravings, feelings of anxiety, depression, and stress. Cronbach's alpha for the scale was acceptable (α = 0.88). The Drug Cravings scale was dichotomous, with a value of 1 if parents reported any drug cravings at a given occasion, and for the remaining subscales, higher scores reflect higher levels of the respective domain.

#### Service Utilization Survey (SUS)

The SUS is a self-report measure of health care and social service utilization within a prescribed period (i.e., monthly). The SUS not only allows comparison across conditions of services being received, but also is a strong assessment of what traditional services include for clients during the WL phase. The SUS was developed by the first author and study consultant to assess service utilization, and has been used across a number of studies (e.g., Franz et al., 2019).

#### FAIR Fidelity

A 15-item measure was developed to evaluate the content (e.g., “My counselor encouraged me to try fun activities with my child”), process (e.g., “My counselor is available to me when I need support”; “I receive FAIR bucks for my success”), and structure (e.g., “My counselor and I spend a lot of our time together out and about”) of FAIR sessions. Parents were asked to rate their level of agreement (1 = *Strongly Disagree*, 5 = *Strongly Agree*) with the series of statements about their counselor. Further psychometric evaluation is needed; however, preliminary IRT-based Rasch measurement models from this trial indicated that the instrument measures a single dimension of fidelity. The primary distinction in parent ratings was between the highest rating of 5 and all lower ratings, and the level of reliability was 0.72 (interpreted consistently with Cronbach's coefficient alpha).

### Tracking Program Costs

As described, the FAIR program is an intensive treatment program, with unbillable activities (e.g., driving) and therefore, challenging to fund under Medicaid. However, given the client base and the point in a parents' life that FAIR is introduced, Medicaid is the most likely payor for such a program. In order to inform the future transportability and scalability of the program into a billable environment, the FAIR program components were tracked and costed under the assumption of a Medicaid-billable environment. Due to the limited traditional services received in this evaluation, a cost-*effectiveness* evaluation was not conducted. However, this project did allow for an evaluation of program costs to understand the capacity and infrastructure needs necessary to yield a financially stable and sustainable program.

### Data Analysis Strategy

Self-reported parental opioid and/or methamphetamine use, parental mental health, parenting risk, and parental stability outcomes had a common data structure, with repeated measurements (level-1) nested within a maximum of 99 participants (level-2). Of note, the SUS and PDR were administered on a monthly basis, and other outcomes were measured at the full assessment battery occasions. The nested data structure was addressed using mixed-effects regression models (Hedeker and Gibbons, 2006) with a random effect for the nesting of repeated measurements within participants. The models were implemented in HLM software (Raudenbush et al., 2013). There were two types of outcome distributions: Continuous (TSI T-scores, BCAP, BDI, PSI, PDR, months at current residence), which were modeled with a Gaussian distribution and restricted maximum likelihood estimation, and dichotomous (methamphetamine use, opioid use, IV drug use, TSI clinical anxiety, TSI clinical levels in any subscale, PDR cravings, paid work, and money spent on drugs), which were modeled with a Bernoulli distribution (logit link) and penalized quasi-likelihood (PQL2) estimation.

To evaluate FAIR, the research design introduced a unique consideration: intervention condition was not restricted to a single status for each participant. Specifically, participants who transitioned from WL to FAIR were in both conditions over time, and as such, intervention condition was time-varying. Because there were relatively few WL phase observations (see section Referrals, Consent, and Randomization), the model was formulated with *FAIR as the reference phase*, and observations in the WL phase were controlled using a single time-varying indicator (i.e., 0 = FAIR, 1 = WL). To test for change during the FAIR phase (i.e., within-group change), the model included a series of time-varying, dummy-coded indicators to differentiate each of the full assessment battery occasions in the FAIR phase (i.e., Month 4, Month 8, Month 16, and Month 24) from the FAIR baseline. Month indicators were used because outcomes were not expected to change at a constant rate over the 2-year follow-up period and occasion-specific change estimates were more useful for evaluating and revising the intervention. The model formulation—with month indicators for the FAIR phase observations only, and a single WL phase indicator—controlled for WL phase observations and tested for a difference between the FAIR baseline and each later occasion in the FAIR phase. For dichotomous outcomes, odds ratios (ORs) and predicted probabilities are reported in text. The ORs reflect change between the FAIR baseline and each later occasion, and the predicted probabilities reflect the estimated score at the respective occasion. FAIR fidelity was evaluated descriptively based on monthly measurements during the FAIR phase.

## Results

As described in the data analysis procedures, outcomes for this FAIR effectiveness trial examined the change over time from baseline for each of the primary treatment targets, controlling for waitlist. Descriptive statistics for self-reported parental substance use, parental mental health, and parenting risk are reported in Table 1 and mixed-effects regression model results are reported in Table 2. These results are followed by a presentation of the cost-related outcomes for service delivery under a Medicaid reimbursement structure.

**Table 1:** Descriptive statistics for substance abuse, mental health, parenting risk, and parental stability outcomes.

	**Waitlist**	**Month 0**	**Month 4**	**Month 8**	**Month 16**	**Month 24**
**Outcome**	***M(SD)/*%**	***M(SD)/*%**	***M(SD)*/%**	***M(SD)*/%**	***M(SD)*/%**	***M(SD)*/%**
**Substance Abuse^a^**						
ASI any methamphetamine use	43%	47%	26%	15%	15%	18%
ASI any opioid use	20%	20%	7%	5%	1%	1%
ASI any IV drug use	5%	22%	7%	4%	4%	1%
PDR drug cravings^b^	51%	54%	35%	40%	28%	32%
**Mental Health**						
TSI anxiety (T-score)	53.16 (10.98)	58.93 (10.60)	53.93 (11.73)	52.59 (10.33)	51.71 (11.20)	50.34 (10.04)
TSI anxiety (clinical)	16%	29%	23%	14%	11%	10%
TSI any (clinical)	36%	60%	41%	43%	39%	38%
BDI (total score)	18.07 (14.50)	19.76 (13.71)	17.01 (14.92)	14.33 (11.9)	16.76 (14.50)	15.27 (13.77)
**Parenting Risk**						
PSI (total)	230.50 (43.06)	236.29 (41.8)	222.89 (47.52)	220.51 (46.23)	217.51 (46.06)	225.77 (41.7)
BCAP (total)	10.53 (5.47)	10.30 (6.04)	9.77 (6.09)	8.52 (5.63)	9.29 (5.89)	8.17 (5.67)
PDR child behavior^b^	5.50 (4.47)	6.75 (4.88)	5.31 (4.22)	4.09 (3.75)	3.93 (3.97)	4.31 (4.68)
PDR parental stress^b^	7.81 (7.13)	9.68 (8.33)	7.11 (6.55)	5.68 (6.06)	5.02 (5.32)	6.10 (8.10)
PDR emotional distress^b^	1.94 (1.49)	1.92 (1.09)	1.78 (1.25)	1.76 (0.99)	1.78 (1.43)	1.69 (1.45)
**Parental Stability**						
Paid for any work this month	55%	32%	33%	38%	44%	51%
Paid for ≥20 work days this month	23%	7%	12%	14%	26%	28%
Months at current residence^c^	1.84 (1.14)	1.48 (1.21)	1.49 (1.16)	1.73 (1.07)	1.63 (1.16)	1.84 (1.08)

*TSI, trauma symptom inventory; BDI, beck depression inventory; PSI, parent stress index; BCAP, brief child abuse potential inventory; PDR, parent daily report. Percentages are reported for dichotomous outcomes. For substance Abuse, Mental Health, and Parental Stability outcome domains, ns across instruments ranged from 44 to 47 during the Waitlist Phase and from 90 to 91 at FAIR Baseline. Across time in the FAIR condition, n was at 81 during Month 4, and ranged from 78 to 80 at Month 8, from 71 to 72 at Month 16, and from 79 to 80 at Month 24. Several Parenting Risk questionnaires were only administered if the parent was currently in contact with their children. Thus, for this outcome domain, ns across instruments ranged from 32 to 47 during the Waitlist Phase. For the FAIR condition, ns across instruments ranged from 65 to 90 at FAIR Baseline, from 56 to 81 at Month 4, from 55 to 80 at Month 8, from 42 to 72 at Month 16, and from 52 to 79 at Month 24*.

a *Participants' self-reported substance use over the past 30 days*.

b *The PDR was administered on a monthly basis. For descriptive purposes, these reports were averaged by parent, and then across parents, for the time period corresponding to each of the major assessment occasions. All observations were included in mixed-effects regression models*.

c *1 = 1 Month or Less, 2 = 2–6 Months, 3 = 7–12 Months, 4 = ≥13 Months*.

**Table 2:** Mixed–effects regression model estimates for all outcomes.

	**Baseline level**			**Change from baseline**											
	**M00**			**M04**			**M08**			**M16**			**M24**		
**Outcome**	**Est**.	***SE***	***p***	**Est**.	***SE***	***p***	**Est**.	***SE***	***p***	**Est**.	***SE***	***p***	**Est**.	***SE***	***p***
**Substance Abuse^a^**															
ASI any methamphetamine use^b^	−0.09	0.29	0.771	−1.29	0.39	0.001	−2.27	0.45	<0.001	−2.28	0.46	<0.001	−2.03	0.43	<0.001
ASI any opioid use^b^	−1.49	0.30	<0.001	−1.18	0.51	0.022	−1.60	0.59	0.007	−2.96	1.05	0.005	−3.03	1.05	0.004
ASI any IV drug use^b^	−1.40	0.31	<0.001	−1.49	0.54	0.006	−2.36	0.69	0.001	−2.31	0.70	0.001	−3.56	1.08	0.001
PDR Drug cravings^b^	0.20	0.23	0.394	−0.92	0.23	<0.001	−1.00	0.20	<0.001	−1.86	0.36	<0.001	−1.44	0.33	<0.001
**Mental Health**															
TSI anxiety (T–score)	58.81	1.11	<0.001	−5.17	1.09	<0.001	−6.84	1.10	<0.001	−8.41	1.14	<0.001	−8.71	1.10	<0.001
TSI anxiety ^b^ (clinical)	−1.09	0.31	0.001	−0.36	0.41	0.378	−1.29	0.46	0.005	−1.68	0.50	0.001	−1.64	0.50	0.001
TSI any ^b^ (clinical)	0.59	0.30	0.050	−1.06	0.37	0.004	−1.07	0.37	0.004	−1.39	0.39	<0.001	−1.25	0.37	0.001
BDI (total score)	19.61	1.42	<0.001	−3.01	1.44	0.037	−5.99	1.44	<0.001	−4.43	1.49	0.003	−4.66	1.45	0.001
**Parenting Risk**															
PSI (total)	235.73	4.64	<0.001	−13.40	4.77	0.005	−14.44	4.82	0.003	−17.99	5.00	<0.001	−9.58	4.91	0.052
BCAP (total)	10.29	0.64	<0.001	−0.92	0.67	0.171	−2.23	0.67	0.001	−2.16	0.70	0.002	−2.57	0.67	<0.001
PDR child behavior	7.07	0.50	<0.001	−1.92	0.40	<0.001	−2.97	0.37	<0.001	−2.66	0.62	<0.001	−2.43	0.57	<0.001
PDR parental stress	10.01	0.81	<0.001	−3.09	0.65	<0.001	−4.31	0.60	<0.001	−3.96	1.00	<0.001	−3.12	0.92	0.001
PDR emotional distress	1.91	0.11	<0.001	−0.22	0.10	0.035	−0.30	0.09	0.001	−0.26	0.16	0.092	−0.25	0.15	0.102
**Parental Stability**															
Paid for any work this month^b^	−0.86	0.28	0.003	0.10	0.36	0.773	0.34	0.36	0.347	0.64	0.36	0.077	0.97	0.35	0.006
Paid for ≥20 work days this month^b^	−3.05	0.48	<0.001	0.78	0.58	0.181	0.97	0.58	0.094	1.90	0.54	0.001	1.97	0.53	<0.001
Months at current residence^c^	1.48	0.12	<0.001	0.02	0.13	0.904	0.24	0.14	0.072	0.21	0.14	0.144	0.40	0.14	0.004

*TSI, trauma symptom inventory; BDI, beck depression inventory; PSI, parent stress index; BCAP, brief child abuse potential inventory; PDR, parent daily report. Confidence intervals (95%) can be calculated as β ± (1.96 × SE). All models controlled for waitlist observations. Parameter estimates for the waitlist term and variance components are available upon request*.

a *Participants' self–reported substance use over the past 30 days*.

b *Indicates a dichotomous outcome*.

c *1 = 1 Month or Less, 2 = 2–6 Months, 3 = 7–12 Months, 4 = ≥13 Months*.

### FAIR Engagement and Service Delivery

Prior to interpreting clinical outcomes, it was key to determine if referred parents received the intervention being studied. Thus, treatment engagement was considered by examining the percentage of parents who engaged with their FAIR counselor, and the percentage that were retained in services. Across all 91 parents who consented to receive treatment (i.e., were either randomized to FAIR or opted to consent to FAIR when their time arrived to transition from the WL condition), 95% (*n* = 86) engaged in services and, of those, 72% completed their recommended treatment. Of note, 17 parents who received FAIR services engaged in more than one treatment attempt before completing the program (this is not atypical for parents involved in FAIR in the real world, and protocols exist, including a “what will be different this time?” analysis, for parents who seek to re-engage after deciding to discontinue).

Monthly, participating parents were asked about their perceptions of FAIR counselor service delivery using the FAIR Fidelity measure. On a scale of 1–5, across counselors, the average FAIR Fidelity rating was 4.6, which remained consistent over the course of the study. Fidelity items that demonstrated the greatest challenge for counselors were process focused: “my counselor could have been more helpful to me as a parent” and “there are things I did not like about this program,” with an average rating of 1.92 and 2.0, respectively (note these items were reversed scored). Counselors appeared competent on adherence items for content: “I am asked to give a urine sample to test for drugs and alcohol” and “my counselor tests me for drug and alcohol use” with consistent ratings of 5. Thus, it was assumed that FAIR was delivered as intended, with even the most challenging items being rated as above average.

### Primary Effectiveness Outcomes

As shown in Table 2, and described below, parents receiving FAIR demonstrated statistically significant improvements in all treatment target areas compared to baseline. Table 1 provides the descriptive analyses for each assessment at each time point.

#### Parental Methamphetamine and Opioid Use

At baseline, referred parents reported substantial substance abuse histories. Across all participants, 69% had previous substance abuse treatment experience (range 1–15 times), 17% reported a previous history of overdose (range 1–5 times), and 22% reported using intravenously currently. On average parents reported using methamphetamine 6.39 days (*SD* = 10.04, range 0–30) in the last 30 days, and an average of 7.38 years (*SD* = 6.51; range 0–30). Parents reported using opioids for an average of 2.08 days (*SD* = 6.21; range 0–30) in the last 30 days and for an average of 3.15 years (*SD* = 4.78; range 0–22).

**Figure 3:**
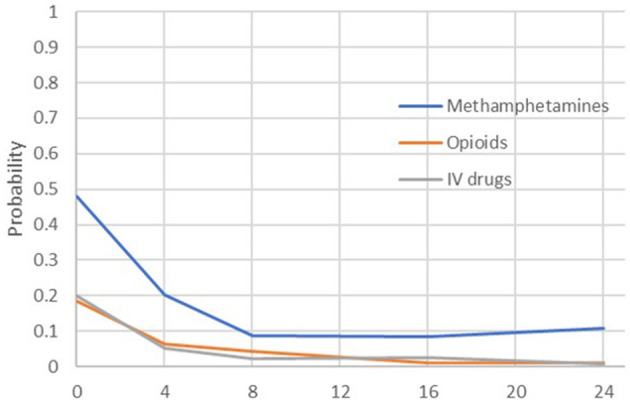
Predicted probabilities of opioid or methamphetamine use by parents across the assessment period.

Across each of the parent-reported methamphetamine and opioid use outcomes, there were statistically significant decreases in reported use between the FAIR baseline and each later assessment occasion (see Table 2). For methamphetamine, the predicted probability of use at baseline was 48%, and over time, this decreased significantly to 20% at Month 4 (OR = 0.28), 9% at Month 8 (OR = 0.10), 9% at Month 16 (OR = 0.10), and 11% at Month 24 (OR = 0.13). For opioids, the baseline rate of use was 18%, which decreased significantly to 6% at Month 4 (OR = 0.31), 4% at Month 8 (OR = 0.20), 1% at Month 16 (OR = 0.05), and 1% at Month 24 (OR = 0.05). For IV drugs, the baseline rate of 20% decreased significantly to 5% at Month 4 (OR = 0.23), 2% at Month 8 (OR = 0.09), 2% at Month 16 (OR = 0.10), and 1% at Month 24 (OR = 0.03). A summary of the self-reported methamphetamine and opioid use outcomes, shown in Figure 3, suggests that both methamphetamine and opioid use showed marked decreases between baseline and 4 months (controlling for waitlist), with incremental decreases and maintenance over time. Of note, each of these outcomes—one occasion at a time and controlling for baseline—were tested for differences between mothers and fathers. No significant effects were found, and therefore, due to the number of analyses run and modest sample size, sex was not included in the subsequent models.

For the PDR drug cravings scale, the baseline rate of any reported problems was 55%, which decreased significantly to 33% at Month 4 (OR = 0.40), 31% at Month 8 (OR = 0.37), 16% at Month 16 (OR = 0.16), and 22% at Month 24 (OR = 0.24).

#### Parental Mental Health

At baseline, parents reported a significant history of experiencing abuse. Across all participants, 70% reported a lifetime history of physical abuse, with 10% reporting experiencing physical abuse in the last 30 days. Half of all participants reported a history of sexual abuse (51%), with 4% indicating an occurrence in the last 30 days. While 79% reported a lifetime history of emotional abuse, 28% reported an occurrence in the last 30 days. Of note, 74% reported having experienced recent emotional distress in the last 30 days related to one or more of these abusive experiences, with 33% reporting daily distress. However, only 22% reported a history of any mental health treatment.

Across mental health outcomes, and with only one exception, there were statistically significant decreases in symptoms between the FAIR baseline and each of the follow-up assessment occasions (see Table 2). Trauma symptoms, as measured by the TSI, indicated Anxiety T-scores decreased from 58.8 at the FAIR baseline to 53.6 at Month 4, 52.0 at Month 8, 50.4 at Month 16, and 50.1 at Month 24. Likewise, the predicted probability of a clinical-level Anxiety score decreased from 25% at baseline to 19% at Month 4 (OR = 0.70; the one non-significant effect), to 8% at Month 8 (OR = 0.27), and to 6% at Months 16 and 24 (ORs = 0.19). Similarly, for a clinical-level score on any TSI scale, the predicted probability decreased significantly from 64% at baseline to 38% at Month 4 (OR = 0.35), 38% at Month 8 (OR = 0.34), 31% at Month 16 (OR = 0.25), and 34% at Month 24 (OR = 0.29). Likewise, symptoms of depression as measured by the BDI decreased significantly across occasions, from 19.6 at baseline to 16.6 at Month 4, 13.6 at Month 8, 15.2 at Month 16, and 14.9 at Month 24. Finally, PDR ratings of emotional distress decreased significantly at Months 4 and 8.

#### Parenting Risk

Table 2 provides the mixed-effects regression outcomes regarding parents' self-reported parenting stress and beliefs as measured by the PSI and BCAP. As seen, parenting stress decreased significantly from 235.7 at baseline to 222.3 at Month 4, 221.3 at Month 8, and 217.7 at Month 16. On the other hand, risk for child neglect did not show significant reductions until Month 8, decreasing from the baseline score of 10.3 to 8.1, with the reduction maintained at 8.1 at Month 16 and 7.7 at Month 24. The PDR ratings of child problem behavior decreased significantly from 7.1 at baseline to 5.2 at Month 4, 4.1 at Month 8, 4.4 at Month 16, and 4.6 at Month 24. The level of stress reported by parents in response to these behaviors also decreased over time, from 10.0 at baseline, 6.9 at Month 4, 5.7 at Month 8, 6.1 at Month 16, and 6.9 at Month 24.

#### Parental Stability

At baseline, almost half of all parents reported their usual living arrangement as being with their partner and children (48%); the remaining parents reported living with family (18%), living alone with their children (13%), or without a stable arrangement (10%). Results for parental stability outcomes are reported in Tables 1, 2. Housing stability did not change significantly at Months 4, 8, or 16, but it did increase significantly at Month 24. The level at Month 24, a predicted score of 1.87, indicates that parents were closer to having lived at their current residence for 7 to 12 months (i.e., a score of 2). For paid work, there were two versions of the outcome: any paid work and full-time work. At baseline, the probability of full-time work was 5%, and this increased significantly at Months 16 and 24 to 24% (OR = 6.71) and 25% (OR = 7.19) respectively. For any paid work, the baseline probability was 30%, and at Month 24, this increased significantly to 53% (OR = 2.64).

### Exploratory Correlations: Associations Across the Four FAIR Components

**Figure 4:**
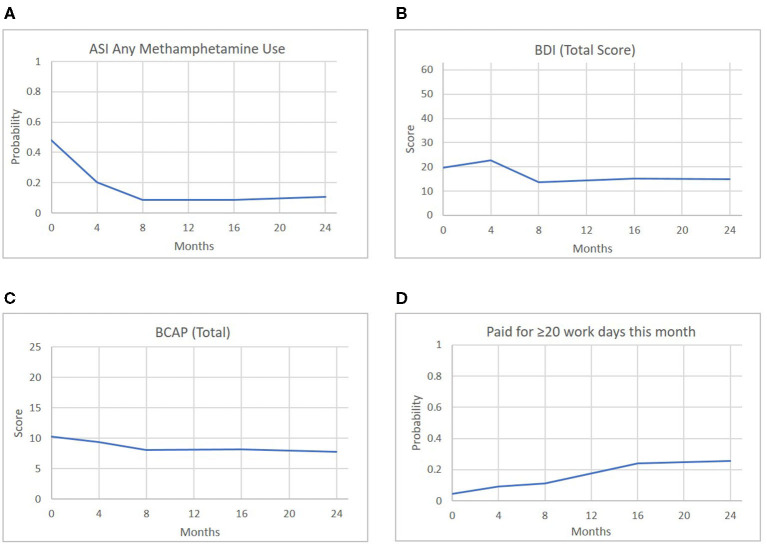
Predicted scores of representative outcomes across the four FAIR domains over time.

As shown in Figure 3, both opioid and methamphetamine use showed marked decreases between baseline and 8 months, with incremental decreases and maintenance over time, and the same pattern held for outcomes related to mental health and parenting risk. To understand these effects—specifically, the degree to which certain outcomes were meaningfully correlated at relevant points in the treatment process—correlations were computed between outcomes from each domain at Month 0 (baseline), Month 8 (around the time that treatment is completed), and Month 16 (~8 months since treatment completion). The selected exemplary variables were methamphetamine use (ASI), depression symptoms (BDI), risk for parental neglect (BCAP), and paid work (Parental Stability). Figure 4 illustrates each outcome across the full 24-month follow-up, and the correlations are reported in Table 3. At baseline, methamphetamine use was not significantly correlated with the other variables. Among the selected variables, the only significant correlation at baseline was between depression and risk for parental neglect. By Month 8, methamphetamine use was significantly associated with depression symptoms, and depression symptoms continued to be associated with parental neglect. By Month 16, methamphetamine use continued to be significantly correlated with depression symptoms, and there was also a significant and positive correlation with parental neglect. Also at Month 16, methamphetamine use, depression symptoms, and parental neglect all had significant, negative correlations with paid work. As shown in Table 3, a number of significant associations exist across all four FAIR treatment domains, highlighting the interrelated symptoms presentation.

**Table 3:** Correlations between select outcomes from the substance abuse, mental health, parenting risk, and parent stability domains at FAIR baseline, Month 8, and Month 16.

**Outcome**	**Methamphetamine**		**Depression**		**Risk for neglect**	
	***r***	***p***	***r***	***p***	***r***	***p***
**FAIR Baseline**						
ASI any methamphetamine use						
BDI (depression total score)	0.14	0.172				
BCAP (risk for neglect total)	−0.01	0.959	0.74	<0.001		
Paid for ≥20 work days this month	−0.07	0.485	−0.19	0.066	−0.10	0.390
**Month 8**						
ASI any methamphetamine use						
BDI (depression total score)	0.47	<0.001				
BCAP (risk for neglect total)	0.19	0.139	0.65	<0.001		
Paid for ≥20 work days this month	−0.17	0.137	−0.22	0.050	−0.05	0.691
**Month 16**						
ASI any methamphetamine use						
BDI (depression total score)	0.59	<0.001				
BCAP (risk for neglect total)	0.32	0.018	0.67	<0.001		
Paid for ≥20 work days this month	−0.25	0.031	−0.28	0.018	−0.30	0.024

### Cost and Reimbursement of FAIR Service Delivery

Table 4 provides the average total cost of a client over the course of treatment. The FAIR team is comprised of a mix of Qualified Mental Health Associates (certified drug and alcohol counselors) and Qualified Mental Health Professionals (who hold a Master's degree or above). In the study's local Medicaid environment, QMHPs are allowed to conduct intake assessments and to complete the interim clinical assessments required by Medicaid. QMHAs are allowed to provide all non-assessment services delivered within the FAIR program, but are reimbursed at a reduced rate. The cost per clinician was calculated (i.e., salary/fringe, phone, mileage, session expenses; $5,680/month) and totaled to the fixed monthly program expenses (i.e., billings and software, supervision, rent, medical director, administrative support) for an average clinician cost of $7,938/month. These figures do not consider additional expenses such as training and turnover costs. Total cost per client estimates were calculated by using an average of true costs for 30 completed cases. Outcomes suggest a cost of $8,000–9,000 per client, over an average treatment length of 8.7 months. As shown in Table 4, monthly treatment costs decreased over the course of FAIR, as level of intervention intensity decreased over time.

**Table 4:** Average cost of treatment per client, per treatment month.

**Month**	**Average client % of FTE**	**Monthly client cost**
1	19%	$1,520
2	16%	$1,280
3	14%	$1,120
4	15%	$1,200
5	12%	$960
6	13%	$1,040
7	10%	$800
8	8%	$640
9	6%	$480
Total		$9,040

The ability to recoup costs through Medicaid reimbursement changed over time due to changes in reimbursement fee schedules by the county insurer. Although at the start of the trial, a modifer was provided for offering services outside of the clinic, with an additional modifier for providing rural services, by the end of the trial both of these additional reimbursement credits were no longer allowable. Thus, to continue to provide services to parents in rural, outlying communities (i.e., nearly half of referred parents), the clinic was required to seek additional funding for 13% of program costs above Medicaid. Table 5 shows an analysis of travel costs for FAIR, with figures adjusted for no-show appointments. As shown, counselors had to travel to settings no more than 20 miles away, 7–10 times, to cover the cost of delivering a single encounter in the most distant locations.

**Table 5:** Travel costs for providing FAIR treatment throughout the catchment area of study clinic.

	**Trip expenses**			**Average trip revenue**			**Profit**	
	**Round trip miles**	**Total travel cost**	**Clinical wage cost**	**Average units billed**	**Average revenue**	Adjusted average revenue^*^	**Adjusted revenue minus expenses**	**Breakeven % rate increase**
**QMHP^a^**								
	84	$132.20	$86.00	6.3	$208.66	$146.06	–$72.14	49.39%
	45	$67.75	$90.11	6.6	$218.59	$153.01	–$4.85	3.17%
	20	$28.92	$51.88	3.8	$125.86	$88.10	$7.30	−8.29%
**QMHA^b^**								
	84	$114.20	$68.01	6.3	$169.79	$118.85	–$63.36	53.31%
	45	$58.75	$71.25	6.6	$184.49	$129.14	–$0.86	0.66%
	20	$25.17	$41.02	3.8	$108.00	$75.60	$9.41	−12.45%

* *Includes the 30% no–show rate for trips made without any billable units*.

a *QMHP, Qualified Mental Health Professional (master's degree or above)*.

b *QMHA, Qualified Mental Health Associate (bachelor's degree with experience)*.

## Discussion

This trial examined the clinical effectiveness and financing required to sustain FAIR—an intensive community-based outpatient program for families involved in the CWS with parental opioid and/or methamphetamine use—when delivered in a real-world community behavioral health clinic.

### FAIR Engagement

Although treatment engagement rates were high at 95%, only 72% completed the program. While this treatment completion rate is less than found in the original randomized clinical pilot where 87% completed treatment (Saldana, 2015), it still exceeds the rates reported across residential (65%) and outpatient (52%) substance abuse treatments across the United States (Stahler et al., 2016) or Family Treatment Drug Courts with parents involved in the CWS (65%; Worcel et al., 2008). This is particularly significant considering the comprehensive and integrated benefits of the FAIR program, in addition to substance abuse treatment. Indeed, as shown throughout the results, parents who received FAIR showed significant improvements in areas related to mental health, parenting, and ancillary stability. Thus, the engagement strategies utilized by FAIR counselors as part of the defined intervention demonstrate strong potential to engage and retain a particularly difficult to engage population.

### FAIR Effectiveness

The overall outcomes from this trial suggest the clinical effectiveness of the FAIR program in addressing all four treatment components targeting the needs of parents referred by the CWS for opioid and/or methamphetamine abuse (Figure 2). Of note, the average length of treatment was 8.7 months, with a 24-Month follow-up (i.e., 15.3 months post-average treatment completion). Therefore, parents who received FAIR were likely to maintain improvements in their substance abuse, mental health symptoms, and parenting risk for over a year after completing treatment. Although only 25% of participants were fathers, the probability of reducing opioid or methamphetamine use did not differ for mothers vs. this small sample of fathers, offering cautious promise of FAIR in providing an effective treatment for either parent referred by the CWS.

At baseline, the majority of parents referred to this study (74%) reported experiencing distress in the last 30 days related to a previous experience of abuse. Of these, 33% reported experiencing daily distress and yet, only 22% of parents reported any history of mental health treatment. Exploratory analyses highlighted the relationship between parental depression, methamphetamine use, risk for child neglect, and employment. These patterns are consistent with conceptualization of the logic behind FAIR (Figure 1), and underscores the need to address the interrelation of all treatment domains to achieve the goal of safe and stable families. Although only a sample of available correlations were detailed, significant non-reported associations were found across a range of variables including other substance use scales, mental health symptoms, and parenting risk indicators, highlighting the overarching need for comprehensive care for families involved in the CWS.

### Waitlist

Although the adapted version of the dynamic wait-listed design was intended to accommodate the ethical concerns of not making an efficacious treatment available to a population in high need, the high rate of participants who accepted the invitation to receive FAIR once a slot became available was not expected. Given that participants initially randomized to WL were encouraged to seek alternative services and maintained the opportunity to receive compensation for their research participation, it was anticipated that a larger portion of the WL sample would have declined FAIR, providing greater opportunity to examine a no-treatment group in addition to waitlist effects. Of the 40 parents initially allocated to WL, 13 reported receiving some level of mental health and/or substance use treatment, but only 8 declined FAIR once it was offered. Thus, only 33% of parents initially randomized to WL engaged in services outside of FAIR, and 80% of parents referred elsewhere preferred to try FAIR even though its level of rigor and commitment was more intensive than traditional outpatient services. This secondary finding reinforces not only that the CWS has identified a need for services specific for families with opioid and methamphetamine abuse, but parents themselves who have open CWS treatment plans desire a needs-specific program. Although the original design failed in providing a large enough sample for rigorous comparisons between groups, this failure highlights the misalignment between the needs of parents and the services that are traditionally available.

Given the limited number of individuals remaining on the waitlist throughout their 24-months participation (*n* = 8), formal analyses were not conducted comparing this group against parents receiving FAIR. Yet, the WL data still offer some value. Across time, parents who remained on the waitlist, opting to receive services elsewhere, showed moderate reductions both in methamphetamine and opioid use at 8 Months, but use was close to baseline for both substances by 24 Months, with associated high levels of cravings and other substance-related problems. Although two individuals reported decreased mental health symptoms, the majority reported relatively unchanged mental health symptoms. One exception was anxiety, which showed a steady increase in severity from baseline to 24 Months. Parenting risk behaviors were inconsistent across this small sample. While these waitlist observations are limited, they offer a preliminary example of the potential trajectories for families with complex needs who do not receive integrated services.

### Services for Families Involved in the Child Welfare System

As described in the results, the baseline functioning of parents referred to the study was notably poor. Parents described extensive periods of methamphetamine and/or opioid use. Less frequent, but still reported, was the use of other illicit drugs including benzodiazepines, cocaine, MDMA, and hallucinogens. In the original feasibility trial of FAIR, the average age of onset of any substance use was 16 years (Saldana et al., 2013). The average age of the current sample was 31.24 years, with a longer reported length of use reported for methamphetamines (7.38 years) than opioids (3.15 years). Several older parents reported up to 30 and 22 years of use for methamphetamine and opioids, respectively. Thus, families presenting to FAIR demonstrate the level of severity of parents who are referred to the CWS who are in need of an array of services.

The FAIR logic model (Figure 1) was developed over a decade ago from a series of qualitative interviews and focus groups with CWS-involved collaborators including workers, legal teams, and parents during the formative development work. The current trial shows that the need for programs like FAIR is as great now as it was at its inception, and also shows that if parents are able to access such services, they might be able to break out of a cycle of high ancillary need. Although indicators of parental stability initially did not change for parents receiving FAIR, by Month 16 they reported increases in days employed and, by Month 24, significant increases in full-time employment and housing stability.

### Cost and Financing of FAIR

The current trial examined a free-standing FAIR program, functioning independently of the research study. As described, the average cost of treating a FAIR parent was ~$9,000 over the course of ~9 months. Though costly, the average cost of methadone maintenance treatment for opioid use disorder alone is $4,700 annually (National Institute on Drug Abuse, 2018), and does not address the other complexities for long-term parental success. Likewise, inpatient addiction treatment costs range between $14,000 and $27,000 for a 30-day treatment (American Addiction Centers, 2021) and may not address the specific needs of parents involved in the CWS. Although a formal cost-effectiveness analysis was not feasible in the current trial, it is hypothesized that future research will find FAIR to be cost-effective relative to the combination of services received by parents as part of their CWS treatment plans.

Federal guidelines establish the base for reimbursement fee schedules and definitions of billable services; however, states and their contracted Medicaid providers operate independently of one another, making it difficult to determine a fixed expectation of costs and reimbursements available. Current CPT codes do not provide reimbursement for services such as FAIR and, as such, individual session activities are billed whenever possible, but unbillable time still remains. As shown in Table 5, these financing challenges limit the ability for programs like FAIR to serve families beyond a prescribed mileage radius without the assistance of additional funding. Thus, such programs also must consider factors such as the geographic range being served in their financial strategies. When such factors are considered, however, and with close financial monitoring, FAIR can be sustained within a community clinic setting.

### Limitations

Despite the strong clinical effectiveness of FAIR found in the current trial, several important limitations should be noted. First, although the dynamic wait-listed design offered a rigorous alternative to traditional randomized clinical trials and was necessary to meet the ethical and CWS needs, it failed to provide the intended goal of having a reasonable sample of parents who remained on the WL for repeated measurement periods, and therefore limited the ability to draw firm comparisons between parents receiving FAIR and those who receive traditional services. This meant that the statistical tests, rather than focusing on differences between groups, focused on within-group change over time, controlling for waitlist. Second, the statistical models tested for change between baseline and each later occasion. This provided targeted tests, but one consequence was that the model assumed all participants completed assessments at the intended timing of each occasion. Alternative formulations (e.g., linear slopes) could address uneven spacing of measurements across participants. Third, the FAIR program being evaluated was a single site, operating in the same county where it was developed. Therefore, the CWS was a part of the intervention development process and was familiar with the program. It is unknown how FAIR might be received in a new community under different CWS conditions. Fourth, due to challenges unrelated to the study at the state DHHS office providing administrative outcomes, data is not yet available to determine FAIR's effectiveness in achieving system-level outcomes such as rates of case closure and child permanency.

### Future Directions

In addition to these outcomes offering promise as a treatment for adults with complex and interrelated problems, they demonstrate the effectiveness of an intervention for one of the most intractable issues facing the CWS, specifically, and public serving systems more generally. The FAIR program has been operating consistently since its inception in 2009, growing steadily from a feasibility trial to an independent program. Indeed, a recent analysis showed the ability for the FAIR program to sustain during the COVID-19 pandemic (Cruden et al., 2021) demonstrating the promise for FAIR to sustain and become recognized within a CWS and service system community.

To help facilitate the possibility of scale-up, implementation strategies were developed to implement FAIR in a new context. Strategies build from those used by the investigative team for implementing other interventions and include an operationalized implementation plan, cost calculator based on findings from the currently described trial, and a training and coaching process. An active effectiveness-implementation trial of an adapted version of FAIR to prevent parental opioid and/or methamphetamine use is evaluating the effectiveness of these strategies in implementing FAIR in nearby counties, as part of the Helping to End Addiction Long-Term initiative (PI: Saldana; UG/H3DA050193). These scale-up efforts, in combination with outcomes from the current trial, underscore the promise for FAIR to be implemented more widely.

As communities across the United States struggle to address the opioid and methamphetamine crises, the FAIR program might offer families access to evidence-based practice in a welcome style. Policy efforts are needed to focus on investing in reimbursement for programs that address the complexities of parental opioid and/or methamphetamine use, and in so doing focus on investing in future generations.

## Data Availability Statement

The datasets presented in this article are not readily available but data might be made available upon request to be analyzed in collaboration with members of the investigative team. Requests to access the datasets should be directed to Lisa Saldana, lisas@oslc.org.

## Ethics Statement

The studies involving human participants were reviewed and approved by Oregon Social Learning Center Institutional Review Board. Written informed consent to participate in this study was provided by the participants' legal guardian/next of kin.

## Author Contributions

LS conceptualized and led this study, developed the intervention and hypotheses, and led the writing of the manuscript. JC developed the study design and data analytic plan, directed primary outcome analyses, and assisted in manuscript preparation including writing and editing. MC conducted all economic analyses and helped with manuscript preparation. ZA conducted primary data analyses under the direction of JC and assisted in manuscript preparation including writing analysis results. HS conducted all preliminary data preparation analyses and participated in manuscript preparation. CP oversaw study procedures and reviewed all study descriptions for accuracy. All authors contributed to the article and approved the submitted version.

## Conflict of Interest

LS is the developer of the FAIR program and initiated the implementation of FAIR at the ODI Clinic. She does not gain financially from this implementation. Further, she was not involved in data collection, management, or analyses or otherwise positioned to manipulate study outcomes. The remaining authors declare that the research was conducted in the absence of any commercial or financial relationships that could be construed as a potential conflict of interest.
